# Purification and Identification of Novel Myeloperoxidase Inhibitory Antioxidant Peptides from Tuna (*Thunnas albacares*) Protein Hydrolysates

**DOI:** 10.3390/molecules27092681

**Published:** 2022-04-21

**Authors:** Bingna Cai, Peng Wan, Hua Chen, Jingtong Huang, Ziqing Ye, Deke Chen, Jianyu Pan

**Affiliations:** 1 Key Laboratory of Tropical Marine Bio-Resources and Ecology, Guangdong Key Laboratory of Marine Materia Medica, South China Sea Institute of Oceanology, Chinese Academy of Sciences, 164 West Xingang Road, Guangzhou 510301, China; bncai@scsio.ac.cn (B.C.); wanpeng@scsio.ac.cn (P.W.); chenhua@scsio.ac.cn (H.C.); huangjingtong21@mails.ucas.ac.cn (J.H.); yeziqing19@mails.ucas.ac.cn (Z.Y.); cdk@scsio.ac.cn (D.C.); 2 Southern Marine Science and Engineering Guangdong Laboratory (Guangzhou), No.1119, Haibin Road, Nansha District, Guangzhou 511458, China; 3 Innovation Academy of South China Sea Ecology and Environmental Engineering (ISEE), Chinese Academy of Sciences, Guangzhou 510000, China

**Keywords:** *Thunnas albacares*, antioxidant peptide, MPO, molecular docking

## Abstract

Antioxidative peptides that inhibit myeloperoxidase (MPO) enzyme activity can effectively defend against oxidative stress damage. The antioxidant peptides from tuna protein were produced using alcalase hydrolysis and purified by ultrafiltration and Sephadex G-15, and the fractions with the highest free radicals scavenging ability and oxygen radical absorbance capacity (ORAC) values were sequenced using HPLC–MS/MS. Fifty-five peptide sequences were identified, 53 of which were successfully docked into MPO. The representative peptide ACGSDGK had better antioxidant activity and inhibition of MPO chlorination and peroxidation than the reference peptide hLF1-11. The docking model further showed intense molecular interactions between ACGSDGK and MPO, including hydrogen bonds, charge, and salt bridge interactions, which occluded the active site and blocked the catalytic activity of MPO. These results suggested that the antioxidant peptide ACGSDGK has the potential to inhibit oxidative stress and alleviate inflammation in vivo because of its inhibitory effect on the MPO enzyme.

## 1. Introduction

Tuna is a marine fish with high economic value, and its demand is increasing worldwide. According to The State of World Fishers and Aquaculture by the Food and Agriculture Organization (FAO), the global tuna catch reached 7.5 million tons in 2018 [1]. Yellowfin tuna (*Thunnus albacares*) is the second most important species of tuna, accounting for approximately 30% of the global catch (FAO). Fish trade produces underutilized fish by-products including trimmings, heads, viscera, frames, skin, fins, and roe, which account for more than 60% of the total biomass [2]. These by-products are good protein resources. The protein content of tuna trimmings is as high as 80.71 g/100 g [3], which makes it a potential resource to develop bioactive peptides to facilitate numerous nutraceutical applications and improve its commercial value. Recently, many studies have shown that antioxidant peptides from fish proteins are easily absorbed and highly cost-effective for human health consumption [4].

Antioxidative peptides in fish proteins play an important role as beneficial functional factors that protect against oxidative stress, which is associated with chronic diseases, such as inflammation, hypertension, cardiovascular disease, cancer, diabetes, and ageing problems [5]. It has been reported that the antioxidant peptides have hydrogen/electron-donating or metal chelating activities, which can terminate the chain reaction or prevent the formation of free radicals [6]. The interaction between antioxidant peptides and free radicals is affected by their structural properties, amino acid composition and locations [7]. Previous studies have shown that hydrophobic amino acids (Leu, Val, Ala, Pro, Phe, Tyr, and Trp) and specific amino acid residues (Cys, Met, Lys and His) can improve the activity of antioxidant peptides [8,9,10,11]. The mechanism of antioxidant peptides can be verified by molecular docking with myeloperoxidase (MPO). Molecular docking is a theoretical method based on bioinformatics for studying the interaction between ligands and receptors and has been widely used for screening and discovering marine active compounds in recent years [12]. MPO is a peroxidase that stimulates oxidative stress in a variety of inflammatory diseases. Inhibition of increased MPO activity can alleviate inflammation associated with diseases [13]. It has been reported that the peptide hLF1-11 (GRRRRSVQWCA) obtained from human lactoferrin can bind to the edge of and within the crevice of the active site of MPO to inhibit its enzyme activity [14]. A peptide DTETGVPT from *Volutharpa ampullacea perryi* [15] and a peptide TEFHLL from Atlantic sea cucumber were also found to have potential MPO inhibitory activity [16]. Therefore, inhibition of MPO enzyme activity or elimination of MPO-derived oxidants is a target for screening antioxidant peptides.

Proteolytic hydrolysis is one of the most widely used methods to prepare antioxidative fish peptides [17]. The type of exogenous proteolytic enzymes used affects the functional properties of peptides. Alcalase has been proven to be among the most efficient proteases for antioxidant peptides and improves the functional, sensory and nutritional properties of foods [18]. Antioxidant peptides from tuna (*T. albacares*) by-products were hydrolyzed with alcalase and demonstrated good activity [19]. The ultrafiltration retentate (<1 kDa) of the tuna dark meat hydrolyzed using alcalase exhibited the strongest DPPH and ABTS radical scavenging activities [20]. Skipjack tuna by-products that were hydrolyzed by alcalase exhibited the highest radical scavenging activity, which might be related to the high content of aromatic amino acid and hydrophobic residues, small molecular residues, and amino acid sequences [21]. However, the structure and mechanism of antioxidant peptides from tuna by-products need to be further studied.

Hence, the purpose of this study was to screen the potential antioxidative peptides that inhibit MPO activity. In brief, the aims of these studies are to optimize the hydrolysis conditions of antioxidant peptides by alcalase, and isolate the antioxidant peptides by ultrafiltration and gel chromatography, and identify the peptide sequence of the antioxidant peptides using HPLC-ESI-QTOF MS/MS. Finally, the antioxidant mechanism of antioxidant peptides was analyzed by molecular docking.

## 2. Results and Discussion

### 2.1. Effect of Enzymolysis Time on the Antioxidant Activity of Hydrolysate

**Figure 1:**
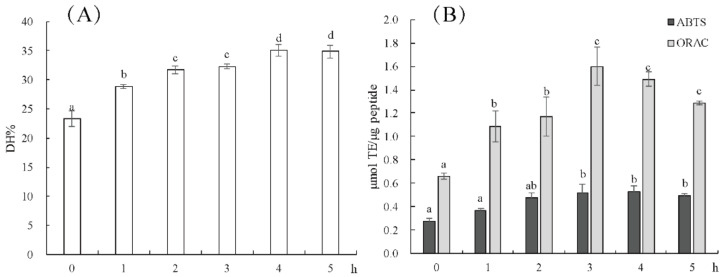
Hydrolysis degree (**A**) and antioxidant activity (**B**) of hydrolysate at different enzymolysis times. Different lowercase letters on the bar indicated significant difference (*p* < 0.05) between groups.

The hydrolysis degree of tuna meat after alcalase hydrolysis for 1–5 h is shown in Figure 1A. The degree of hydrolysis of hydrolysate at 4 h was 35.08 ± 1.08%, and there was no significant difference between 5 h hydrolysis and 4 h hydrolysis, indicating that enzymatic hydrolysis was basically complete at 4 h. It is reported that the DH of tuna viscera protein and head protein by alcalase is different, which are 20% [22] and 9.24% [19], respectively. These differences in DH are due to differences in the amino acid composition of substrate proteins. The comparison of antioxidant activities of the hydrolysates at different times is shown in Figure 1B. With the extension of hydrolysis time, the ABTS^+^ scavenging activity and ORAC values of tuna hydrolysates increased and stabilized at 3 h. The hydrolysates at 3 h had better antioxidant capacity, although the content of small molecular peptide was lower than that of hydrolysates at 4 h and 5 h. In other words, the antioxidant capacity is related not only to the molecular weight of the product, but also due to the amino acid composition, peptide sequence and structure, which is consistent with the report of Zhang et al. [16]. Thus, the TPA3 was further purified and studied for antioxidant peptide screening.

### 2.2. Antioxidant Activity of TPA3 Ultrafiltration and Gel Separation Fractions

**Figure 2:**
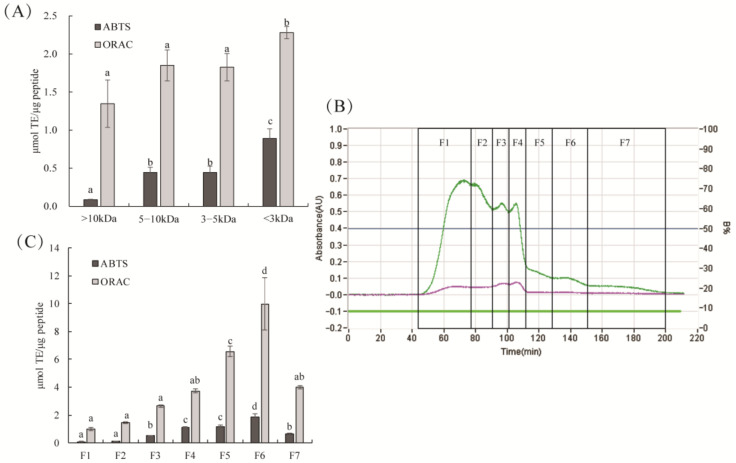
The antioxidant activity of ultrafiltration fractions (**A**). Fraction <3 kDa with the strongest antioxidant activity was separated by Sephadex G-15 gel (**B**). Seven fractions were collected, and their antioxidant activity was determined by ABTS and ORAC assays (**C**). Different lowercase letters on the bar indicated significant difference (*p* < 0.05) between groups.

The antioxidant activity of the ultrafiltration fractions is shown in Figure 2A. The hydrolysate fraction with an MW < 3 kDa showed the strongest ABTS^+^ scavenging ability and ORAC values, which was consistent with the higher antioxidant activity of hydrolysates with smaller molecular weights reported previously [23]. Small peptides with low molecular weights can interact with free radicals more effectively and more easily through the intestinal barrier [6]. These results are similar with previous study that the lowermost molecular weight fraction (<3 kDa) from yellowfin tuna protein hydrates had the highest antioxidant activity [24]. Then, seven fractions were obtained by Sephadex G15 chromatography (Figure 2B). The ABTS^+^ scavenging activity and ORAC values of the F6 fraction were significantly higher than those of the other fractions (Figure 2C, *p* < 0.05). The ABTS and ORAC methods are based on a hydrogen atom transfer reaction [25]. This indicated that F6 might have a stronger hydrogen atom transferability than the other fractions. Moreover, the ORAC value of purified F6 was four times higher than that of the ultrafiltration crude component, which may be due to the increased concentration of active peptide after fractionation. These results indicated that more antioxidant peptides with relatively low molecular weight were enriched in the F6 fraction.

### 2.3. Identification and Screening of Potential Antioxidant Peptides in Silico

There were 55 peptides identified from the F6 fraction, and their information is listed in Table 1. The molecular weight of the peptides ranged from 470 to 1130 Da, with 7–14 amino acid residues. Additionally, most of the peptides had good water solubilities. Due to the nonspecific alcalase, the amino acid residues on the C-termini of the identified peptides were more diverse, such as Asp, Ala, Lys, Leu, Phe and Met. Previous studies found that antioxidant peptides generally contained hydrophobic amino acids, such as Val, Leu, Ala, Phe and Pro, which can enhance the interaction between antioxidant peptides in the lipid phase of water to promote radical scavenging [26]. These residues were found in almost all 55 identified peptides.

In addition, it has been reported that peptides rich in aromatic amino acids such as Trp, Tyr and Phe, sulfur-containing amino acid residues such as Cys and Met, and basic amino acid residues such as Lys and His show higher biological activity by electron transfer or hydrogen atom transfer [6,27,28]. Aromatic amino acid residues present in antioxidant peptides can change the free radicals to stable molecules by electron donation [29]. Cys and Met residues can scavenge free radicals and react with peroxides due to the sulfur-containing group [30]. There were 35 peptides in the F6 fraction found to contain aromatic and/or sulfur- and/or basic-containing amino acid residues, which contributed to its high radical scavenging capacity. Antioxidant peptides reported from mung bean meal protein and Atlantic Sea cucumber in the literature have also been found to contain these characteristic amino acid residues [16,31].

In addition, docking analysis was performed to further screen the potential peptides that inhibit MPO. The reference peptide GRRRRSVQWCA was successfully docked in a crevice containing the haem pocket, which was identified as the catalytic site of MPO [14,32]. Therefore, based on the catalytic site, 96.4% of peptides in the F6 fraction successfully docked with the MPO enzyme (Table 1), indicating potential antioxidant activity in vivo. Generally, a higher (-) CDOCKER energy value suggests better binding affinities between the ligand and the receptor. Thus, the first six peptides with higher (-) CDOCKER energy than the reference peptide GRRRRSVQWCA were considered better potential inhibitors of the MPO enzyme (Table 1). These peptide sequences essentially contain basic amino acids (Lys, His or Arg). The reference bioactive peptide GRRRRSVQWCA also contained 36.36% of the basic amino acid Arg, which was important for binding to the crevice of the active site of MPO [14].

### 2.4. The Activity of Synthesized Peptides

To verify our prediction, the first six peptides in Table 1 were synthesized, and their antioxidant activity and inhibition of MPO were evaluated. As shown in Table 2, the peptide sequences KFCSGHA and ACGSDGK had higher free radicals scavenging activity and inhibited chlorination and peroxidation of the MPO enzyme better than the other peptide sequences from octopus. Among them, the ABTS and ORAC antioxidant values of ACGSDGK were higher than those of the reference peptide GRRRRSVQWCA, with significant differences, while the IC_50_ value of inhibiting MPO peroxidation was 0.0179 mM, lower than that of GRRRRSVQWCA, indicating that ACGSDGK displayed greater antioxidant activity. ACGSDGK had an IC_50_ value of the MPO chlorination similar with that of GRRRRSVQWCA. In addition, peptide KFCSGHA exhibited ABTS and ORAC values and IC_50_ values for MPO peroxidation that were similar with those of GRRRRSVQWCA, but presented a higher IC_50_ value for MPO chlorination than that of GRRRRSVQWCA. These two bioactive peptides shared the same amino acid residue as GRRRRSVQWCA with Cys, which was essential for interfering with MPO due to its cysteic acid group [14].

**Figure 3:**
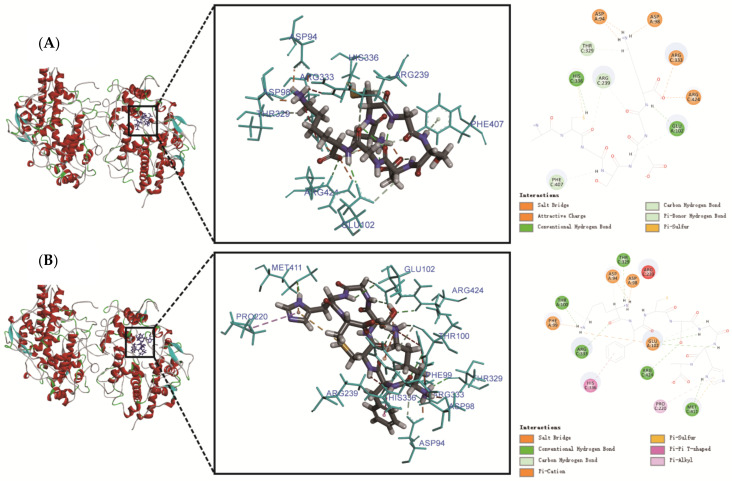
Three-dimensional and 2D structure graphs of the molecular interactions between peptide ACGSDGK (**A**) and MPO, as well as between peptide KFCSGHA (**B**) and MPO.

More accurate molecular interactions between MPO and the bioactive peptides were performed to further elucidate the exact binding sites and mode, as displayed in Figure 3. The 3D structure graphs showed that the two peptides were well docked into a crevice containing the haem pocket of MPO. On the side of the haem unit, histidine 336, histidine 95, arginine 239, and glutamate 242 may be directly involved in the catalytic mechanism [32]. As shown in Figure 3 and Table 3, ACGSDGK and KFCSGHA docked well and stably into the catalytic active site of MPO. The MPO docking graphs of the two peptides were like previous result of antioxidant peptides from *kinema* [33], and both blocked the entrance to the active cavity of MPO. Twelve hydrogen bonds and five electrostatic interactions formed between ACGSDGK and MPO (Figure 3A). In particular, the Cys residue in ACGSDGK formed a hydrogen bond and Pi-sulfur with His336 and two hydrogen bonds with Arg239 on the C chain of MPO, which are the catalytic active sites of MPO. It has been found that antioxidant peptides can form hydrophobic interactions with one of the catalytic residues (Arg239) of MPO [34]. Pi-sulfur interaction facilitates charge transfer between ligand and receptor [35]. In addition, several other favorable interactions (salt bridge and attractive charge) exist between non-catalytic residues and ACGSDGK (Figure 3A). These non-covalent contacts formed between MPO and the peptide can maximize the binding affinity of the ligand to the target [33]. This finding indicated that ACGSDGK has good inhibition activity of chlorination and peroxidation of MPO. MPO is secreted by activated leukocytes at sites of inflammation and produces strong oxidants such as hypothalamus acids (HOX, X = Cl, Br) [36]. HOX species readily react with sulfur-containing amino acids, such as Cys and Met. In other words, the presence of Cys was necessary to inhibit the hypochlorite/hydroxyl radicals produced by MPO.

In addition, 14 hydrogen bonds, two hydrophobic interactions and four charge interactions were observed between KFCSGHA and MPO (Figure 3B and Table 3). Although KFCSGHA had a lower interaction energy than ACGSDGK, its inhibition activity was weaker than that of ACGSDGK. The reason may be that only a Pi-Pi T-shaped interaction was observed between KFCSGHA and His336 in MPO. In contrast, ACGSDGK binds to more catalytic active sites of MPO. There were also two peptides containing the basic amino acid Lys at the C-terminus or N-terminus, which bind to the crevice of the active sites by electrostatic interactions with Asp94 and Asp98 on the A chain of MPO. Together, the binding of ACGSDGK and KFCSGHA to these amino acid residues interrupted the hydrogen bond, salt bridge and charge interactions in the active site, which are necessary for MPO catalysis. Therefore, ACGSDGK and KFCSGHA showed good binding affinity to MPO catalytic residues, which can effectively inhibit the activity of MPO and play an important role in regulating oxidative stress.

## 3. Materials and Methods

### 3.1. Materials

Yellowfin tuna trimmings (*T. albacares*) were obtained from Guangdong Xingyi Marine Bioengineering Co., Ltd. (Guangdong, China). Alcalase protease (400,000 U/g) was purchased from Pangbo Enzyme Co., Ltd. (Guangxi, China). DL-Dithiothreitol (DTT), 2,2-azinobis (3-ethylbenzothiazoline-6-sulfonic acid) diammonium salt (ABTS), fluorescein, 2,2′-azobis (2-methylpropion-amidine) dihydrochloride (AAPH) and linoleic acid were purchased from Sigma Co. (St. Louis, MO, USA). Ultrafiltration membranes (VIVAFLOW 200 TM, 10,000, 5000 and 3000 MWCO PES) were obtained from Sartorius Stedim Biotech GmbH (Goettingen, Germany). All other chemical reagents used in the study were analytical grade.

### 3.2. Preparation of TPA Protein Hydrolysates

Yellowfin tuna trimmings were mixed with 3 volumes of distilled water (*v*/*w*, mL/g), and the mixture was thoroughly homogenized. The solution was adjusted with 1 M NaOH solution to pH 8.0, and 3000 U/g alcalase was added. Enzymatic hydrolysis was performed in a thermostatic oscillator with 100 r/min at 50 °C for 5 h, and the sample was taken at an interval of 1 h and was terminated in a boiling water bath for 10 min and then centrifuged at 30,000× *g* for 30 min. The supernatant was lyophilized and defined as TPA1, TPA2, TPA3, TPA4 and TPA5, respective. Then, the hydrolysis degree and antioxidant activity of TPA were analyzed.

### 3.3. Determination of Hydrolysis Degree Using the O-Phthaldialdehyde (OPA) Method

The degree of hydrolysis (DH) was investigated according to the OPA methods [37]. Serine standard (50 mg) was diluted in 500 mL deionized water. Deionized water was used as a blank. The sample contained 82% protein and was dissolved with deionized water at different concentrations (6.25–1000 µg/mL). Aliquot of 0.4 mL of serine standard, blank and sample were mixed with OPA reagent (3 mL). The mixture was shaken for 5 s, and the absorbance was determined at 340 nm after 2 min.

(1) $$Serine-NH_{2}=\frac{OD_{sample}-OD_{blank}}{OD_{standard}-OD_{blank}}\times \frac{0.9516meqv/L\times 0.1\times 100}{X\times P},\;h=\frac{serine-NH_{2}-\beta }{\alpha }\;DH=\frac{h}{h_{tot}}\times 100\%$$

where *X* = g sample; *P* = protein % in sample; and the values of constants α, β and $h_{tot}$ were 1.00, 0.40 and 8.6, respectively, for fish.

### 3.4. Purification of TPA

The TPA with the highest antioxidant activity was separated using ultrafiltration membranes with molecular weight cut-offs of 10,000, 5000 and 3000 Da (Sartorius Stedim Biotech GmbH Goettingen, Germany). Four fractions were obtained and freeze-dried for antioxidant assays. The ultrafiltration fraction with the highest antioxidant activity was then eluted with a Sephadex G-15 gel fraction column (5.0 × 80 cm) with distilled water at a flow rate of 5 mL/min. The absorbance was detected at 220 nm and 280 nm with automatic chromatography (EZ Purifier III, Shanghai Lisui Chemical Engineering Co., Ltd., Shanghai, China). Seven gel fractions were lyophilized for the subsequent antioxidant assay.

### 3.5. Antioxidant Assay

#### 3.5.1. ABTS Radical Scavenging Ability Assay

The ABTS radical scavenging ability was investigated using the method of Zheng et al. [38] with minor modification. Briefly, ABTS solution was prepared in darkness using 2.45 mM potassium persulfate and 7 mM ABTS ammonium salt for 16 h. Before use, the ABTS solution was diluted with 50 mM of PBS (pH 7.4) to make its absorbance value reach 0.700 ± 0.020 at 734 nm. After the mixture 150 µL of diluted ABTS resolution and 50 µL of sample added to a 96-well microplate, and was recorded by a microplate reader (Enspire, PerkinElmer, Singapore) at 734 nm at 30 °C after 30 min.

#### 3.5.2. Oxygen Radical Absorbance Capacity (ORAC) Assay

The ORAC values were measured following the method of Wu et al. [39] with a slight modification. All reagents and samples were prepared with PBS (75 mM, pH 7.4). In brief, 50 µL of sample, PBS or Trolox and 150 µL of 78 nM fluorescein solution in a 96-well plate were mixed and incubated at 37 °C for 15 min in a microplate reader, and subsequently added 25 µL of AAPH (221 mM). The fluorescence was measured every 2 min for 120 min with excitation at 485 nm and emission at 535 nm at 37 °C.

An equivalent volume of Trolox (6.25–100 µM) and 50 mM PBS (pH 7.4) was used instead of the sample as the standard antioxidant and blank. The ABTS radical scavenging ability and ORAC values were, respectively, calculated by the slope of Trolox regression curve. Final ABTS radical scavenging ability and ORAC values were expressed as μmol TE/µg peptide with at least three dependent experiments.

### 3.6. Identification of Peptide Sequence

The gel fraction with the strongest antioxidant activity was identify by liquid chromatography-tandem mass spectrometry (HPLC–MS/MS) utilizing a Bruker Q-TOF Premier mass spectrometer (Bruker Daltonic Inc., Billerica, MA, USA) coupled with electrospray ionization (ESI). Sample was subjected to YMC-Triart C18 (250 × 4.6 mm, 5 µm) column. Eluent A consisted of 0.1% formic acid in ultra-pure water, and eluent B consisted of 0.1% formic acid in acetonitrile. The elution program for peptide separation was as follows: 0–15 min, 0–3% B; 15–45 min, 3–25% B; 45–47 min, 25–90% B; and 47–51 min, 90–10% B, with a flow of 1.0 mL/min. The ESI system was operated in positive mode with a capillary voltage of 3.8 kV and scan range of m/z 100–2000. Mascot Distiller v2.4.2.0 software (Matrix Science, Boston, MA, USA) and UniProt database were used to analyze the data, and sequences with a confidence >95% were considered reliable.

### 3.7. Molecular Docking

Molecular docking between the human MPO enzyme (PDB ID: 3F9P) and each bioactive peptide was performed by Discovery Studio 2019 according to He et al. [15] with minor modifications. The identified peptides were constructed and energy-minimized using ChemOffice 20.0 software. The MPO structure was treated by removing water and small molecular ligands, adding hydrogen atoms to the CHARMM force field, and minimizing the energy with the Prepare Protein program in Discovery Studio. The binding site was defined from the receptor cavities using the CDOCKER protocol. The biological peptide hLF1-11 (GRRRRSVQWCA) derived from human lactoferrin [14] had reported inhibitory activity against MPO and was used as a reference. Finally, the potential peptides that inhibited MPO enzyme activity were screened out according to the docking energy.

### 3.8. Peptide Synthesis

The biological peptides and GRRRRSVQWCA were synthesized using the FMOC solid-phase procedure by ChinaPeptides Co., Ltd. (Shanghai, China). The purity of the synthetic peptides was verified to be higher than 95% by analytical HPLC equipped with an API150-ESI mass spectrometry system.

### 3.9. Analysis of MPO Inhibitory Activity

The inhibitory effect of peptides on MPO was estimated using an MPO inhibitor screening assay kit (Abnova, Taiwan), which was used to assess in both the peroxidation and chlorination activities of MPO. Peroxidation assay utilizes the peroxidase component of MPO. During the reaction, hydrogen peroxide reacts with 10-acetyl-3,7-dihydroxyphenoxazine (ADHP) to yield resorufin, a highly fluorescent compound. The chlorination assay utilizes non-fluorescent 2-[6-(4-aminophenoxy)-3-oxo-3H-xanthen-9-yl]-benzoic acid (APF), which is selectively cleaved by hypochlorite to produce fluorescein. The 4-aminobenzhydrazide was used as positive control. The MPO inhibitory activity was determined according to the kit instructions.

### 3.10. Statistical Analysis

All data were expressed as means ± standard deviation (SD) with at least three dependent experiment and analyzed by one-way analysis of variance (ANOVA) with the Bonferroni multiple range tests using IBM SPSS 22.0 software. *p* < 0.05 was considered as significant.

## 4. Conclusions

In this study, two peptides, ACGSDGK and KFCSGHA, with potent inhibition activity of MPO were obtained from tuna protein hydrolysate using conventional antioxidant-guided fractionations and molecular docking methods. ACGSDGK showed a greater antioxidant activity and inhibition of chlorination and peroxidation of MPO than KFCSGHA and GRRRRSVQWCA. This activity may be due to the two regions of ACGSDGK; that is, the Lys at the N-terminus binds to the crevice of the MPO active site by electrostatic interactions with Asp94 and Asp98 on the A chain of MPO, and Cys forms a hydrogen bond and salt bridge with His336 and two hydrogen bonds with Arg239 at the catalytic active site of MPO. These results indicated that the antioxidant peptide ACGSDGK has the potential to inhibit oxidative stress and reduce inflammation in vivo. However, the antioxidant and anti-inflammatory effects of ACGSDGK should be further verified with animal experiments.

## Figures and Tables

**Table 1:** Information on the reference peptide and identified peptides contained in F6.

No.	Peptide Sequence	Length	Mass (Da)	Hydrophobic (%)	(-)CDOCKER Energy	Special Amino Acid (%)			
						Hydrophobic (Leu, Val, Ala, Pro, Phe)	Aromatic (Tyr, Trp, Phe)	Sulfur (Cys, Met)	Basic (Lys, His, Arg)
Ref	GRRRRSVQWCA	11	1374.59	27.27	139.238	18.18	9.09		36.36
1	DVSDLDAD	8	848.3399	37.50	148.631	37.50			
2	GDAFDKA	7	722.3463	42.86	147.225	42.86	14.29		14.29
3	KFCSGHA	7	748.3569	28.57	146.132	28.57	14.29	14.29	28.57
4	DASHGHSG	8	766.2994	12.50	145.510	12.50			25.00
5	GELALKD	7	744.4017	42.86	144.636	42.86			14.29
6	ACGSDGK	7	636.2664	14.29	143.951	14.29		14.29	14.29
7	GGGGGYE	7	595.2754	0.00	139.034			14.29	14.29
8	ASCGGRR	7	706.37	14.29	138.127	14.29			28.57
9	GGYGFGGGAG	10	798.3297	20.00	137.873	20.00		14.29	
10	SGRSAVVS	8	761.4031	37.50	133.948	37.50	20.00		
11	CTMGNGA	7	652.3063	28.57	131.608	14.29			
12	DLSSNVTV	8	833.4131	37.50	129.646	37.50		28.57	
13	GFAGGDGL	8	692.3129	37.50	129.560	37.50			
14	SSQSSGY	7	714.282	0.00	127.791		12.50		
15	ASAAADQ	7	633.312	57.14	127.737	57.14	14.29		
16	DVDTRAYF	8	985.4505	37.50	127.695	37.50			12.50
17	CTIANGG	7	634.3504	28.57	127.565	14.29	25.00		
18	FGVGGGN	7	607.333	28.57	125.585	28.57		14.29	
19	SAINGYF	7	770.3599	42.86	125.186	28.57			
20	ASGCCCK	7	670.2618	14.29	124.649	14.29	28.57		
21	GYGGGVS	7	595.2602	14.29	122.442	14.29		42.86	14.29
22	FGTGGAG	7	565.2591	33.33	121.529	28.57	14.29		
23	AACGGLN	7	604.279	42.86	121.129	42.86	14.29		
24	GFGGGAGSV	9	707.3239	33.33	120.649	33.33		14.29	
25	GPTGHDGAPG	10	864.3726	30.00	120.500	30.00	11.11		
26	AADILDA	7	688.412	71.43	120.067	57.14	10.00		10.00
27	IATVGGG	7	573.2186	42.86	119.743	28.57			
28	FVAAALW	7	777.4286	100.00	119.420	85.71			
29	AGGPAGE	7	558.312	42.86	119.045	42.86	28.57		
30	FGVGGNG	7	606.3189	28.57	117.189	28.57			
31	AIGGAGG	7	501.2955	42.86	116.584	28.57	14.29		
32	GIGGAGG	7	487.2794	28.57	116.110	14.29			
33	ALSCGIW	7	748.3914	57.14	115.762	28.57			
34	SSSGGHG	7	587.2299	0.00	115.684		14.29	14.29	
35	AAAMALW	7	733.445	100.00	112.952	71.43	14.29		14.29
36	GATGGAG	7	489.2353	28.57	112.748	28.57	14.29	14.29	
37	AAAIVVS	7	630.37	85.71	111.837	71.43			
38	ASAAAMN	7	634.3488	71.43	111.757	57.14			
39	GAGGGLS	7	517.2932	28.57	110.742	28.57			
40	AGCSGPH	7	627.3033	28.57	110.149	28.57			
41	AGGSGPAG	8	572.2554	37.50	110.149	37.50		14.29	
42	AGPAGGSG	8	572.2554	37.50	107.837	37.50	14.29	14.29	14.29
43	PTGHLGE	7	709.3279	28.57	107.319	28.57			
44	AGGPTGE	7	588.322	28.57	106.705	28.57	14.29		14.29
45	YSSWMMDA	8	1005.357	50.00	103.526	12.50			
46	GPGGSTA	7	546.496	28.57	101.149	28.57	25.00	25.00	
47	GETGPSGP	8	700.3027	25.00	100.992	37.50			
48	APGGAGG	7	485.2632	42.86	100.016	42.86			
49	GGGGAPG	7	471.2485	28.57	97.938	28.57			
50	MYPGIAD	7	765.3367	57.14	95.151	28.57			
51	PDPAGSG	7	599.2851	42.86	94.751	42.86	14.29	14.29	
52	PASGANG	7	572.2554	42.86	93.382	42.86			
53	GAAGPPGP	8	622.3074	62.50	74.040	62.50			
54	AAGGGCA	7	505.2212	42.86	-	42.86			
55	GFAGSMFGSGAL	12	1100.496	50.00	-	41.67			

**Table 2:** MPO inhibition and antioxidant activity of synthesis peptides.

Peptide Sequence	IC_50_ Value of the MPO Chlorination Assay (mM)	IC_50_ Value of the MPO Peroxidation Assay (mM)	ORAC (µmol TE/mg Peptide)	ABTS (µmol TE/mg Peptide)
GRRRRSVQWCA	0.0272	0.0328	0.77 ± 0.04 ^a^	1.28 ± 0.19 ^a^
DVSDLDAD	0.2957	-	0.00 ± 0.00 ^b^	0.00 ± 0.00 ^b^
KFCSGHA	0.1079	0.0403	0.82 ± 0.05 ^a^	1.30 ± 0.11 ^a^
GDAFDKA	-	-	0.04 ± 0.02 ^b^	0.00 ± 0.00 ^b^
DASHGHSG	-	-	0.15 ± 0.01 ^c^	0.00 ± 0.00 ^b^
GELALKD	0.1221	-	0.15 ± 0.04 ^c^	0.00 ± 0.00 ^b^
ACGSDGK	0.0323	0.0179	0.96 ± 0.09 ^a^	1.78 ± 0.12 ^b^

“-” the IC value of the MPO assay was more than 0.5 mM. Data in the same row with different letters (a, b, c) indicated significant differences at *p* < 0.05.

**Table 3:** Amino acid residues involved in the interactions between bioactive peptides and MPO from predicted binding models.

Peptide Sequence	Interactions	Number	Interaction with A Chain of MPO	Interaction with C Chain of MPO
ACGSDGK	H-bond	12	Asp98, Glu102 (2), Asp94 (2)	Arg239 (2), Thr329, His336, Phe407, Arg424 (2)
	Charge	5	Asp94 (2), Asp98	Arg333, Arg424
	Other	1	-	His336
KFCSGHA	H-bond	14	Asp94 (2), Asp98, Thr100 (2), Glu102 (4)	Thr329, Arg333 (2), Met411, Arg424
	Hydrophobic	2	-	Pro220, His336
	Charge	4	Asp94, Asp98, Phe99, Glu102	-
	Other	1	-	Met411

## Data Availability

Not applicable.

## Sample Availability

Samples of the compounds are available from the authors.
